# Integration of Single-cell and Bulk Transcriptome Analyses Unravels a Macrophage-based Gene Signature for Prognostication and Treatment in Triple-negative Breast Cancer

**DOI:** 10.7150/ijms.120593

**Published:** 2026-01-01

**Authors:** Yuan Huang, Yuan Yu, Yufei Zhu, Qianhui Lu, Ziwen Zhang, Xiaojia Wang, Xiaowei Wang, Yabing Zheng

**Affiliations:** 1 Department of Breast Medical Oncology, Zhejiang Cancer Hospital. Hangzhou Zhejiang, 310022, PR China.; 2 Department of Colorectal Surgery, Sir Run Run Shaw Hospital, School of Medicine, Zhejiang University, Hangzhou 310020, PR China.

**Keywords:** triple-negative breast cancer, macrophages, signature, prognosis, immunotherapy, malignant behaviors

## Abstract

**Objective:** As a dominant component within the tumor microenvironment, macrophages exert essential roles in nearly all aspects of triple-negative breast cancer (TNBC). This work explored macrophage-associated signature genes for prognostication and treatment for TNBC.

**Methods:** Single-cell (GSE180286) and bulk transcriptome profiles (TCGA-TNBC, GSE96058 and GSE45255) were analyzed by multiple computational approaches. The expression of signature genes was verified in tumor tissues and paracancerous normal tissues from patients with TNBC (n=5), HER2^+^ breast cancer (n=5), and HR^+^ breast cancer (n=5) through immunofluorescence and Western blot. Additionally, gene expression was examined in breast cancer cells (MDA-MB-231, and MCF-7) and mammary epithelial cells (MCF10A) using RT-qPCR and Western blot. Following RNA interference or overexpression, CCK-8, wound scratch and Transwell assays were performed. To assess model robustness, 1000 iterations of Bootstrap resampling were performed to calculate optimism-corrected performance metrics; calibration curves were generated via the rms package. Decision Curve Analysis (DCA) was conducted to evaluate the clinical decision-making value.

**Results:** A single-cell map of the microenvironment in non-TNBC and TNBC was depicted. At both the single-cell and bulk levels, macrophages exhibited a higher abundance in TNBC versus non-TNBC. A macrophage-based gene signature (CTSD, CTSL, ELK4, HSPA8, XRCC4) was developed, with a high-risk score predicting poorer outcomes. This signature demonstrated reliable performance in external validation, particularly for one-year survival (AUC > 0.9). Bootstrap analysis corrected the original AUC from 0.706 to 0.739 (optimism=-0.033, difference <5%), and AUC values from 1000 resamplings concentrated in 0.70-0.75 (standard deviation=0.018). External validation confirmed the signature's ability to reliably predict patient prognosis, especially one-year survival. High-risk patients showed greater responsiveness to immunotherapy. The aberrant expression of CTSD, CTSL, ELK4, HSPA8, and XRCC4 in TNBC and non-TNBC was validated both *in vivo* and *in vitro*. Knockdown of XRCC4 attenuated malignant behaviors of MDA-MB-231, MCF-7, and MCF10A cells, whereas overexpression of CTSD, CTSL, and HSPA8 produced the opposite effect.

**Conclusion:** Altogether, a novel macrophage-based gene signature was proposed for estimating survival outcomes and treatment responses in TNBC. The aberrant expression of these signature genes contributes to tumor malignant progression. Our findings offer valuable insights for future clinical research involving macrophages in TNBC.

## Introduction

Triple-negative breast cancer (TNBC) remains the most lethal form of breast cancer, accounting for 15%~20% of all breast malignancies^1^. TNBC cells are highly aggressive and lack of hormones and growth factor receptors^2^. Due to the absent or low expression of estrogen receptor, human epidermal growth factor receptor 2 (HER2), and progesterone receptor, TNBC shows resistance to hormones and endocrine treatment^3^. In comparison to other breast cancer types, TNBC remains the most challenging owing to its greater heterogeneity, higher risk of distant metastases and recurrence, and the lack of validated treatment targets^4^. Currently, chemotherapy is utilized as the major treatment approach against TNBC^5^. With the development of immunotherapy in solid tumors and the recognition of TNBC's immunogenicity, immunotherapy has attracted increasing attention^6^-^8^. For improving the survival outcomes of TNBC patients, identifying novel predictive biomarkers for immunotherapy are challenging^9^. Moreover, due to poor therapeutic responses, there is an urgent need for novel therapeutic targets and predictive biomarkers for prognosis in TNBC.

Myeloid cells, especially macrophages, are the major components within the tumor microenvironment of TNBC^10^. The regulatory mechanisms by which macrophages impact nearly all aspects of TNBC have been extensively studied. For instance, HLF modulates ferroptosis, progression and chemoresistance of TNBC via activation of cancer cell-macrophage communication^11^. Chemotherapy in combination with macrophage suppression increases the abundance of T cells and B cells, leading to durable regression in TNBC^12^. OTUD5-induced deubiquitination of YAP in macrophages contributes to M2 phenotype, which favors TNBC development^13^. To date, only a few macrophage-associated prognostic models have been conducted for TNBC patients, and none of which have been implemented in clinical practice^14^-^16^. Notably, existing models have significant limitations^14^-^16^: Ye et al.^14^ developed a signature based on three gene ratios (PCDH12/ELP3, PCDH12/MSRA, FAM160B2/MSRA) that applies exclusively to TNBC patients receiving adjuvant radiotherapy and fails to predict overall survival (OS) or disease-free survival (DFS) in non-radiotherapy cohorts; Su et al.^15^ constructed a five-gene (CD79A, CXCL13, IGLL5, LHFPL2, PLEKHF1) model that relies on clinical N staging data and focuses solely on chemotherapy sensitivity, with no relevance to immunotherapy response. These limitations underscore the need for a standalone, multi-functional macrophage-related signature for TNBC. In this work, we integrated single-cell and bulk transcriptome data to establish a gene-only macrophage signature that does not rely on clinical staging or treatment type. We further validated its robustness via Bootstrap optimism correction and calibration curves, and evaluated its clinical utility via DCA. Additionally,* in vitro* experiments confirmed the aberrant expression of the signature genes and demonstrated their impact on tumor malignancy.

## Materials and Methods

### Single-cell and bulk transcriptome data acquisition

Raw single-cell RNA sequencing (scRNA-seq) data from four primary TNBC specimens were obtained from the GSE180286 dataset^17^. Three TNBC cohorts with bulk transcriptome profiling and clinical features were obtained from The Cancer Genome Atlas (TCGA) (n=115), GSE96058 (n=3409) 18 and GSE45255 (n=95)^19^.

TNBC sample selection criteria:

TCGA-BRCA: ER(-), PR(-), HER2(-) (confirmed by IHC or molecular typing); complete OS data (follow-up ≥1 month); exclude duplicate patient samples (e.g., TCGA.BH.A18V.01A/06A, latter excluded); final n=115.

GSE96058: Sample annotations explicitly labeled 'TNBC' or 'triple-negative breast cancer'; complete OS data; exclude metastatic or non-primary breast cancer samples; final n=3409.

GSE45255: ER(-), PR(-), HER2(-) (molecular confirmation); complete DFS data; no preoperative neoadjuvant therapy; final n=95.

### Inclusion/exclusion criteria for TNBC samples

TCGA-BRCA Cohort:

Original size: n=1108.

Inclusion: ① IHC/molecular typing confirmed ER(-), PR(-), HER2(-); ② Complete OS data (follow-up ≥1 month); ③ Primary TNBC.Exclusion: ① Duplicate patient samples (e.g., TCGA.BH.A18V.01A/06A, excluded the latter); ② Missing ER/PR/HER2 or OS data.Final size: n=115.

GSE96058 Cohort:

Original size: n=3492.

Inclusion: ① Sample annotations labeled "TNBC" or "triple-negative breast cancer"; ② Complete OS data; ③ Primary tumors.Exclusion: ① Metastatic samples; ② Missing survival status; ③ Non-human samples.Final size: n=3409.

GSE45255 Cohort:

Original size: n=101.

Inclusion: ① ER(-), PR(-), HER2(-) (molecular confirmation); ② Complete DFS data; ③ No neoadjuvant therapy.Exclusion: ① Preoperative chemotherapy/radiotherapy; ② DFS follow-up <3 months.Final size: n=95.

GSE58812 Cohort (Ye et al., 2021 Training Set):

For reference, we applied the following criteria: ① TNBC with adjuvant radiotherapy; ② Complete MFS data. Final size: n=102 (used to verify the consistency of our data processing with their methodology).

Single-cell RNA-seq (GSE180286): ① Batch correction: Seurat v4.0.5 SCTransform (batch variable: "sample source"); ② Normalization: NormalizeData (log2 transformation); ③ Cell filtering: Mitochondrial genes >10%, ribosomal genes <10%, or detected genes <200 → excluded.

Bulk Transcriptomics (TCGA, GSE96058, GSE45255): ① Batch correction: sva v3.44.0 ComBat (batch variable: "sequencing platform/batch ID"); ② Normalization: TCGA (FPKM→TPM); GEO (RMA background correction via affy v1.74.0→TPM); ③ Missing value imputation: k-nearest neighbor (k=5, impute v1.70.0).

### Quality control and preprocessing of scRNA-seq data

By using DropletUtils toolkit 20, empty droplets were identified and removed. Based on Scater toolkit^21^, cells with a mitochondrial gene proportion greater than 10% and a ribosomal gene proportion less than 10% were further excluded. The filtered scRNA-seq data were normalized using Seurat toolkit^22^.

### Principal component analysis (PCA), cell clustering and annotation

The top 2000 highly variable genes were screened utilizing Seurat toolkit, and their expression profiles were linearly scaled, followed by PCA. Next, principal components (PCs) with large standard deviations were chosen, which were subsequently used for cell clustering. Uniform manifold approximation and projection (UMAP) was subsequently applied^23^,^24^. Marker genes in each cell cluster were determined based on the criteria of an average log2 fold change (FC) ≥0.1, cell population expression ratio ≤0.25, and adjusted p≤0.05.

### Immune infiltration analysis

CIBERSORTX^25^ was used to generate labels based on the identification results of single cells as reference expression matrix. In accordance with bulk expression matrix, the proportion of identified cells in each sample was calculated.

### Cell-cell communication

Cell-cell interactions were evaluated using the CellChat package^26^ by analyzing ligand-receptor pairs. The cell-cell communication networks were visualized through Cytoscape software^27^.

### Functional enrichment analysis

Gene Ontology (GO) or Kyoto Encyclopedia of Genes and Genomes (KEGG) pathway enrichment analyses were performed using the clusterProfiler package^28^. KEGG pathways were visualized via pathview web^29^. Gene set enrichment analysis (GSEA)^30^ was employed to identify gene sets with significant differences between groups.

### Differential expression analysis

SCENIC computational approach^31^ was utilized for guiding the identification of transcription factors. Differentially expressed transcription factors were screened between TNBC and non-TNBC macrophages via limma method under the criteria of p ≤0.05 and |t| ≥2^32^. In addition, differentially expressed genes (DEGs) between the groups were selected under the threshold of |log2FC| ≥0.585 and q ≤0.05.

### Least absolute shrinkage and selection operator (LASSO) analysis

Differentially expressed transcription factors and DEGs related to TNBC macrophages were selected for univariate cox regression analysis via survival package. Genes with p ≤0.05 were included for LASSO. TCGA TNBC samples were randomized into training or test cohort. By executing glmnet^33^, signature genes were selected. The risk score was calculated based on regression coefficients combined with the expression levels of the signature genes. Low- or high-risk patients were defined under the median risk score. The LASSO model was externally verified in the GSE96058 and GSE45255 cohorts.

### Bootstrap optimism correction

1000 iterations of Bootstrap resampling (R boot package) were performed: (1) Generate 1000 'pseudo-training sets' (sampling with replacement from the training cohort); (2) Reconstruct the model and calculate the AUC for each pseudo-set; (3) Compute optimism = mean pseudo-training AUC-original training set AUC; (4) Corrected AUC = original AUC-optimism.

### Decision Curve Analysis (DCA)

DCA was conducted in the training cohort (R dca.R script) to calculate net benefit across threshold probabilities (0%-100%), with 'Treat All'/'Treat None' as baselines.

### Genetic mutation evaluation

Somatic mutation data and cancer-testis antigen (CTA) number information of TNBC samples were acquired from the TCGA dataset. Through implementing maftools package, somatic mutation was evaluated and visualized^34^.

### Treatment response estimation

T-cell inflamed score^35^, TIDE score^36^ as well as expression of immune checkpoint molecules^35^ were separately computed for reflecting the response to immunotherapy. Based on the GDSC2 database, IC50 values of drugs were estimated to predict drug response using the oncoPredict package^37^.

### Cell culture

Normal human mammary epithelial cells (MCF10A) as well as human breast cancer cells (MDA-MB-231, and MCF-7) from the Cell Bank of Type Culture Collection of the Chinese Academy of Sciences (China) were cultured in Dulbecco's Modified Eagle Medium (Gibco, USA) with 10% fetal bovine serum (Gibco) and 1% penicillin-streptomycin in a 5% CO_2_ atmosphere at 37 °C.

### Real-time quantitative PCR (RT-qPCR)

Total RNA isolation was isolated using RNAiso Plus reagent (Takara, China), and complementary DNA was synthesized with HiScript III RT SuperMix reagent (Vazyme, China). The primers used were as follows: CTSL, 5'-CTTTTGCCTGGGAATTGCCTC-3' (forward primer), 5'-CATCGCCTTCCACTTGGTC-3' (reverse primer); CTSD, 5'-TGCTCAAGAACTACATGGACGC-3' (forward primer), 5'-CGAAGACGACTGTGAAGCACT-3' (reverse primer); ELK4, 5'-TGGACCTCTAATGATGGGCAG-3' (forward primer), 5'-AGGCTTGTTCTTGCGAATCCC-3' (reverse primer); XRCC4, 5'-ATGTTGGTGAACTGAGAAAAGCA-3' (forward primer), 5'-GCAATGGTGTCCAAGCAATAAC-3' (reverse primer); HSPA8, 5'-ACCTACTCTTGTGTGGGTGTT-3' (forward primer), 5'-GACATAGCTTGGAGTGGTTCG-3' (reverse primer); GAPDH, 5'-ACAACTTTGGTATCGTGGAAGG-3' (forward primer), 5'-GCCATCACGCCACAGTTTC-3' (reverse primer). RT-qPCR was conducted via ChamQ Universal SYBR qPCR Master Mix (Vazyme). The relative mRNA levels were calculated using the 2^-ΔΔCt^ method.

### Western blot

Total protein extractions were performed using RIPA buffer (Cell Signaling Technology, USA), followed by protein quantification with a BCA reagent (Cell Signaling Technology). Protein was separated through SDS-PAGE, with subsequent transference onto PVDF membrane (Millipore, Germany). Following blockade in 5% BCA (Yeasen, China) and incubation with specific antibody against CTSL (1/2000; 27952-1-AP; Proteintech, China), CTSD (1/2000; 21327-1-AP; Proteintech), XRCC4 (1/1000; SC-271087; Santa, USA), HSPA8 (1/500; SC-7298; Santa) and GAPDH (1/2500; 60004-1-Ig; Proteintech). The bands were developed via enhanced chemiluminescence detection kit (Yeasen).

### Patients and samples

Tumor tissues and paracancerous normal tissues were collected from patients with TNBC (n=5), HER2^+^ breast cancer (n=5), and hormone receptor (HR)^+^ breast cancer (n=5) who underwent surgery in Zhejiang Cancer Hospital between 2022 and 2023. All patients were diagnosed by pathologists, and had not received any treatment before surgery. Following the Declaration of Helsinki, ethical approval for the use of human tissues was obtained by the institutional research ethics committee of Zhejiang Cancer Hospital. Written informed consent was obtained from by each patient.

### Immunofluorescence (IF)

Tissue samples were fixed, embedded in paraffin and sliced. Tissue slices were treated with 0.05% trypsin and 3% H_2_O_2_, and blocked using 10% goat serum for 1 h at room temperature. After rinsing with PBS, the slices were incubated with primary antibody against CD11b (1/200; 21851-1-AP; Proteintech), CTSL (1/200; 27952-1-AP; Proteintech), CTSD (1/200; 21327-1-AP; Proteintech), XRCC4 (1/100; SC-271087; Santa, USA), HSPA8 (1/100; SC-7298; Santa) overnight at 4 °C and secondary antibodies for 20 min at 37 °C. DAPI was utilized for nuclear counterstaining. Photographs were acquired under a fluorescence microscope (Nikon, Japan). The images were analyzed through AI digital pathological quantitative analysis software (Visiopharm, China).

### Transfection

The transfection of small interfering RNAs (siRNAs), overexpressing plasmids, and negative controls was carried out using Lipofectamine 2000 transfection reagent (Invitrogen) following the manufacturer's protocol.

### Cell viability assay

Cells were seeded into 96-well plates (2×10^3^ cells/well). Cell Counting Kit-8 (CCK-8) (Yeasen, China) was used for determining cell viability in accordance with the manufacturer's protocols. Absorbance was measured at 450 nm at the indicated time points.

### Wound scratch assay

Cells were seeded into a 6-well plate and cultured until confluent, and the monolayer cells were scraped in a straight line utilizing a 10 µL pipette tip. Next, the plate was washed by PBS to remove detached cells. Photographs were acquired at 0, or 24 h after scratching under an optical microscope (Olympus, Japan).

### Transwell invasion assay

1×10^5^ cells were suspended in serum-free medium and seeded into the upper chamber precoated with Matrigel (BD, USA). The lower chamber contained medium supplemented with 20% FBS. After incubating for 48 h, the invaded cells on the lower surface were fixed by methanol (Beyotime, China), followed by 0.1% crystal violet (Beyotime) staining. Non-invading cells remaining on the upper surface were removed with cotton swabs. The invasive cells were photographed using an optical microscope and counted in randomly chosen fields.

### Statistical analysis

All analyses were performed using R software (version 4.0.3) or GraphPad Prism (version 9.0.1). Differences between two groups was assessed using Student's t test or one-way analysis of variance. Correlation analyses were conducted via Pearson test or Spearman test. Survival curves of overall survival (OS) or disease-free survival (DFS) were generated using the Kaplan-Meier approach, with log-rank test. Receiver operator characteristic curves (ROCs) were plotted using the pROC package. P≤0.05 was considered statistically significant.

## Results

### Single-cell and bulk transcriptome analyses unravel cellular heterogeneity in TNBC

**Figure 1:**
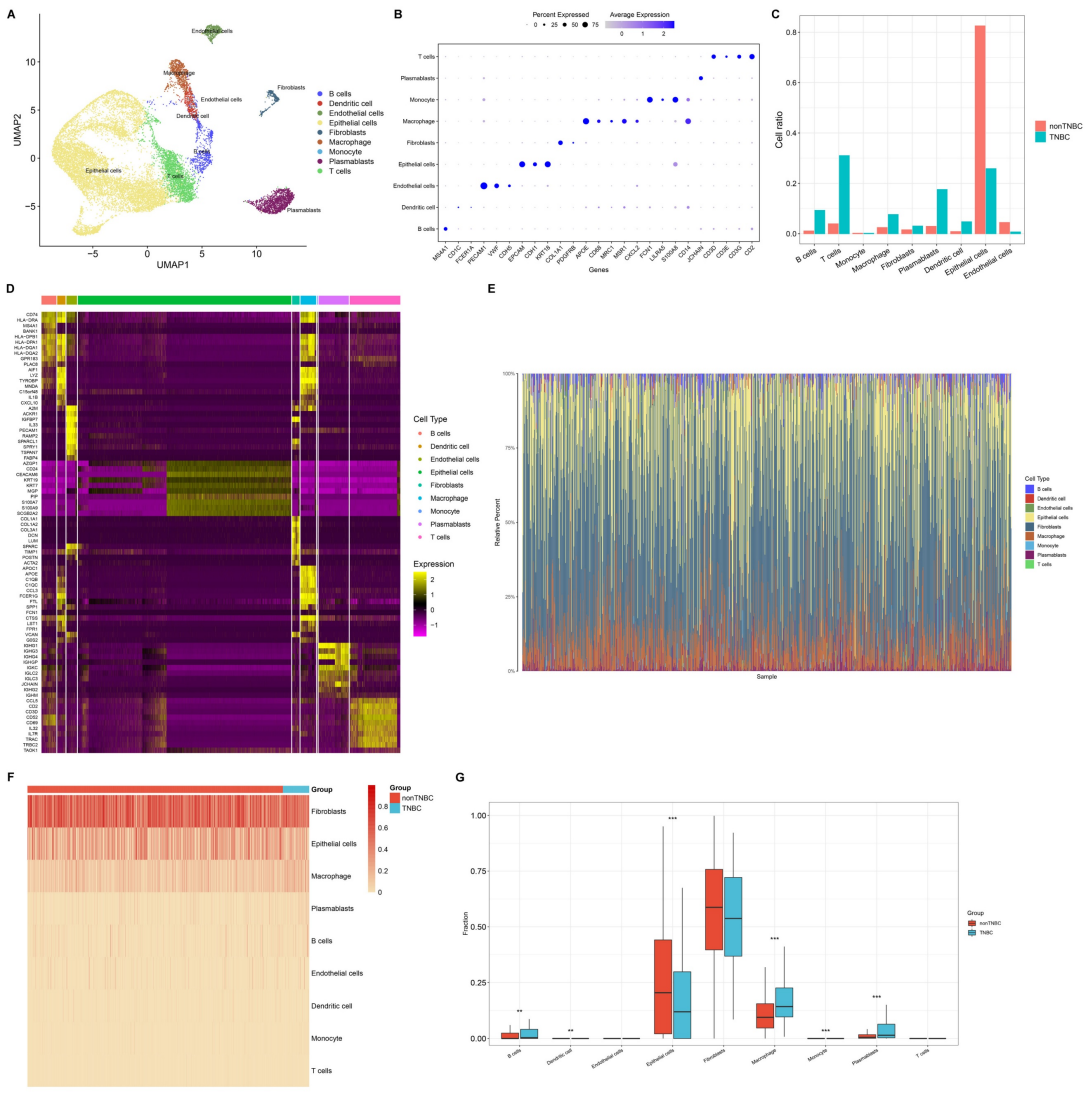
Single-cell and bulk transcriptome analyses unravel cellular heterogeneity in TNBC. (A) UMAP mapping the identified cell populations based on scRNA-seq data. (B) The expression of marker genes across diverse cell populations. (C) Cell proportion of each population in single-cell TNBC and non-TNBC samples. (D) Top 10 novel marker genes for diverse cell populations. (E) The relative cell abundance of identified cell populations in bulk TNBC and non-TNBC tissues. (F, G) Comparison of cell proportions for each population between bulk TNBC and non-TNBC tissues. **p≤0.01; ***p≤0.001.

This study reconstructed a single-cell landscape of TNBC using scRNA-seq data from four primary TNBC specimens. Firstly, low-quality single cells and empty droplets were removed, resulting in the retention of 2599 / 3267 cells in sample GSM5457199, 3872 / 4161 cells in sample GSM5457205, 3755 / 4064 cells in sample GSM5457208, and 6233 / 7521 cells in sample GSM5457211 (**Supplementary figure 1A-L**). Next, the retained scRNA-seq data were scaled based on principal component analysis (PCA), selecting nine principal components (**Supplementary figure 2A-D**). Using UMAP approach, the selected single cells were clustered into 14 clusters, revealing significant cellular heterogeneity between TNBC and non-TNBC (**Supplementary figure 3A-C**). Also, marker genes for each cell cluster were determined (**Supplementary figure 3D, E**). Combining these with known cell type markers, nine distinct cell populations were classified: B cells (n=698), dendritic cells (n=387), endothelial cells (n=495), epithelial cells (n=9984), fibroblasts (n=350), macrophages (n=732), monocytes (n=32), plasmablasts (n=1412), and T cells (n=2369) (**Figure 1A**). The marker genes were specifically expressed in their respective populations: MS4A1 for B cells; CD1C, and FCER1A for dendritic cells; PECAM1, VWF, CDH5, SELE, and CD34 for endothelial cells; EPCAM, CDH1, and KRT18 for epithelial cells; COL1A1 and PDGFRB for fibroblasts; APOE, CD68, MRC1, MSR1, and CXCL2 for macrophages; FCN1, LILRA5, and S100A8 for monocytes; JCHAIN for plasmablasts; CD3D, CD3E, CD3G, and CD2 for T cells (**Figure 1B**). These cell populations differed significantly between TNBC and non-TNBC, with TNBC showing higher proportions of B cells, dendritic cells, fibroblasts, macrophages, plasmablasts, and T cells, and the lower proportions of endothelial cells and epithelial cells in TNBC versus non-TNBC (**Figure 1C**). We also identified novel marker genes for each cell population (**Figure 1D**). Additionally, bulk transcriptome profiles of TNBC specimens from the TCGA dataset were analyzed. Using CIBERSORTx, a reference matrix of cell markers was established based on scRNA-seq data, allowing estimation of relative cell proportions in bulk tissues (**Figure 1E**). Consistent with the scRNA-seq results, macrophages were found at higher proportions in bulk TNBC tissues compared to non-TNBC tissues (**Figure 1F, G**). Thus, macrophages are actively involved in the TNBC microenvironment.

### Cell-cell interactions within the TNBC and non-TNBC microenvironment

**Figure 2:**
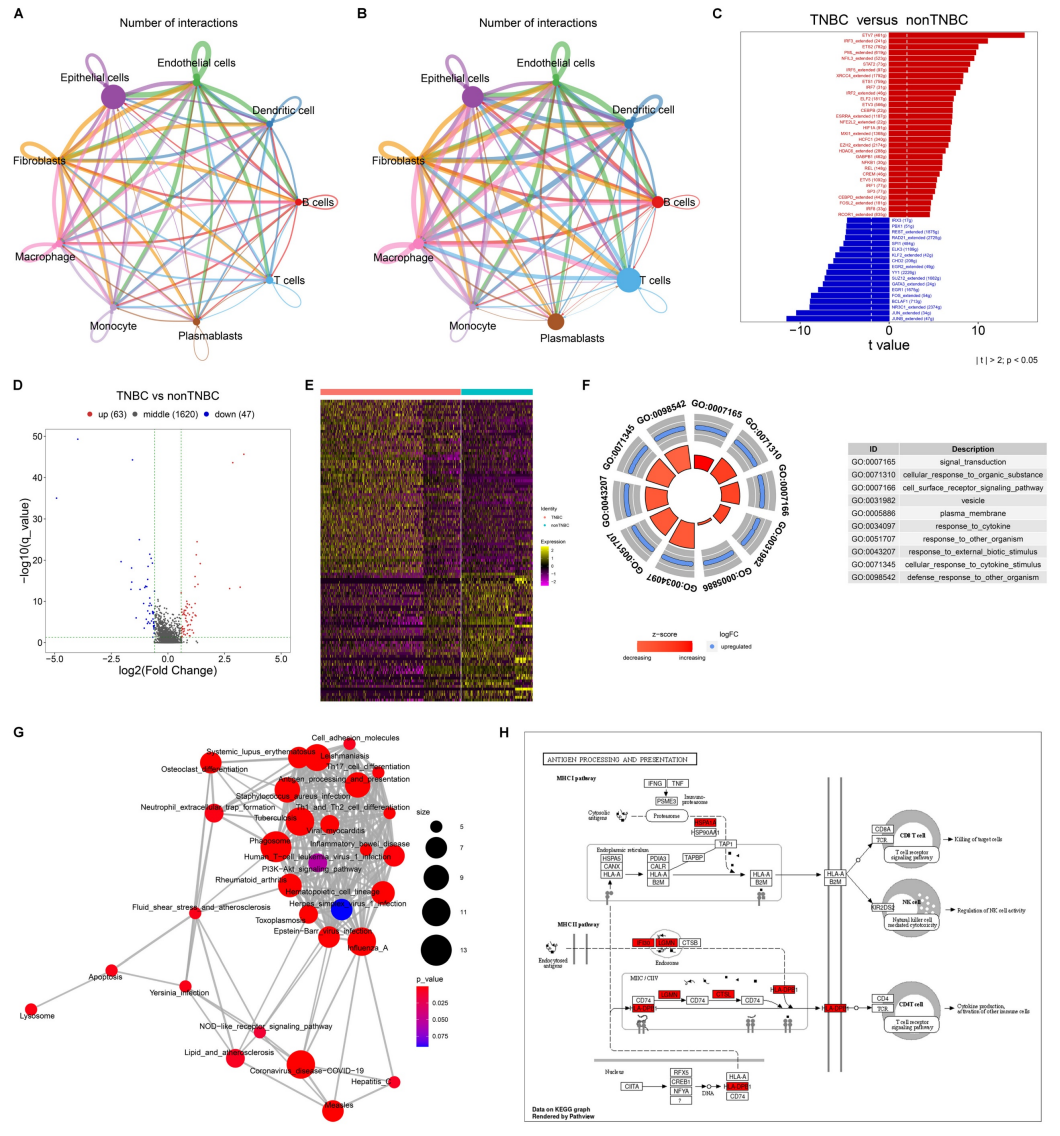
Cell-cell interactions and transcriptional activity of macrophages in TNBC. (A, B) Cell-cell interaction networks in (A) non-TNBC and (B) TNBC samples. (C) Differentially expressed transcription factors in TNBC macrophages versus non-TNBC macrophages. (D, E) DEGs between TNBC macrophages and non-TNBC macrophages. (F, G) The main GO and KEGG pathways enriched by the DEGs. (H) Antigen processing and presentation pathways enriched by the DEGs.

Next, we evaluated cell-cell interactions based on ligand-receptor pairs in non-TNBC and TNBC, respectively. In comparison to non-TNBC, more active cell-cell interactions were observed in TNBC, especially macrophages with other cell populations (**Figure 2A, B**).

### Transcriptional activity of macrophages in TNBC

A total of 104 transcription factors showed differential expression in TNBC macrophages compared to non-TNBC macrophages (**Figure 2C; Supplementary table 1**), suggesting their potential role in transcriptionally regulating macrophage activity in TNBC. In addition, 110 DEGs were determined between TNBC and non-TNBC macrophages (**Figure 2D, E**), with 63 genes upregulated (**Supplementary table 2**) and 47 downregulated (**Supplementary table 3**) in TNBC macrophages versus non-TNBC macrophages. These DEGs were primarily associated with signal transduction, cell surface receptor signaling pathway, response to cytokine, cellular response to cytokine stimulus, etc. (**Figure 2F**). In addition, immune-related pathways were notably enriched among the DEGs, especially antigen processing and presentation (**Figure 2G, H**). Altogether, the functional enrichment analysis highlights the significance of the DEGs in modulating transcriptional activity of macrophages within the TNBC microenvironment.

### Construction of a novel macrophage-relevant prognostic signature for TNBC

**Figure 3:**
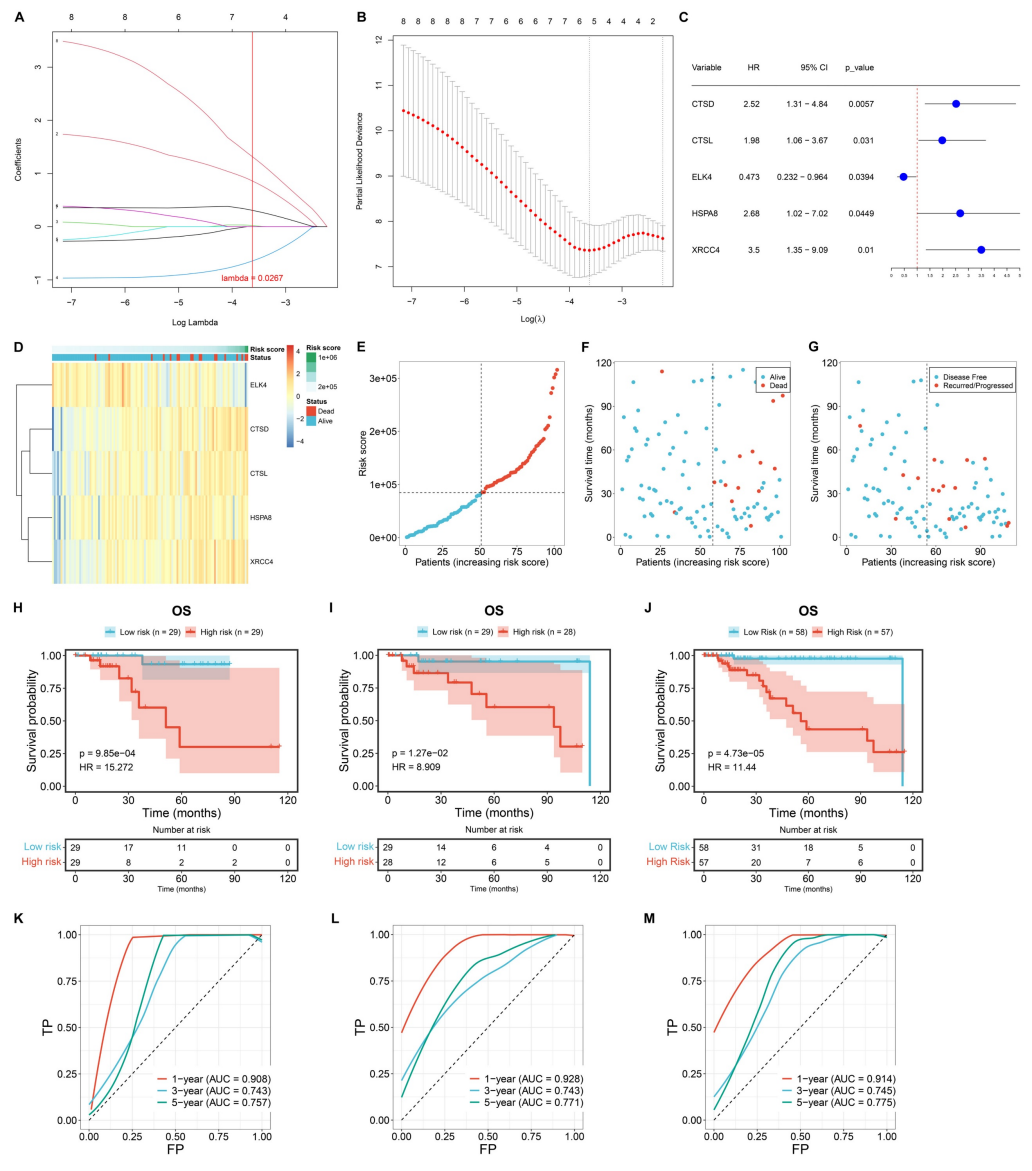
Construction of a novel macrophage-based prognostic signature for TCGA TNBC. (A) Coefficient profiling in the LASSO analysis. (B) Results of 10-fold cross-validation. (C) Univariate-cox regression results of the identified signature genes with TNBC survival. (D) The expression of the signature genes along the risk score. (E) Distribution of risk scores among TNBC cases. (F, G) Distribution of alive/dead or disease-free/recurred/progressed status along the risk score. (H-J) OS probability of low- or high-risk patients in the training, test, and entire cohorts. (K-M) One-, three-, or five-year ROCs based on the prognostic model.

The differentially expressed transcription factors and DEGs associated with TNBC macrophages were included for univariate-cox regression analysis. Consequently, eight genes exhibited significant associations with TNBC prognosis (p≤0.05), composed of C12orf60, CTSD, CTSL, ELK4, FCGR2A, FOLR2, HSPA8, and XRCC4. These genes were then used to construct a LASSO model. TCGA TNBC samples were randomly divided into training and test cohorts. In the training cohort, LASSO analysis was executed for selecting signature genes with regression coefficient ≠ 0. Under the lambda minimum = 0.0267. As a result, five signature genes were eventually selected: CTSD, CTSL, ELK4, HSPA8, and XRCC4 (**Figure 3A, B**). The macrophage-based prognostic signature was established using the following formula: risk score = 0.859575100676907 * CTSD expression + 0.0210700891980921 * CTSL expression + (-0.644138418956012) * ELK4 expression + 0.307340530719732 * HSPA8 expression + 1.31660312733179 * XRCC4 expression (**Figure 3C, D**). Patients with risk scores above the median were classified as high risk, while those with risk scores at or below the median were classified as low risk (**Figure 3E**). The high-risk group exhibited a higher incidence of death or recurrence/progression compared to the low-risk group (**Figure 3F, G**). Survival analysis demonstrated the significantly shorter OS time for high-risk patients in the training cohort (**Figure 3H**). Such survival difference was validated in both the test and entire cohorts (**Figure 3I, J**). In addition, ROCs were plotted to assess the predictive efficacy of the model. In the training (**Figure 3K**), test (**Figure 3L**), and entire (**Figure 3M**) cohorts, the model demonstrated excellent predictive accuracy for one-year survival (AUC>0.9), with an original AUC of 0.706, optimism of -0.033, and a corrected AUC of 0.739 (difference <5%). 1000 resampled AUCs concentrated in 0.70-0.75 (SD=0.018; Supplementary Figure 4). To further evaluate the clinical decision-making value of the model, Decision Curve Analysis (DCA) curves were plotted for 1-year, 3-year, and 5-year survival outcomes in the training cohort (TCGA TNBC, n=115). Meanwhile, "Treat All" (treating all patients) and "Treat None" (treating no patients) were used as baseline controls for clinical decision-making (Supplementary Figure 5-7).

The results are as follows:

1-Year Survival Decision-Making: Within the threshold probability range of 25% (the most commonly used interval in clinical practice), the net benefit of our model reached 0.02 to 0.03, which is several times higher than that of the "Treat None" strategy. This demonstrates that decisions based on our model can significantly reduce both "excessive intervention" (for low-risk patients) and "missed intervention" (for high-risk patients).

(2) 3-Year Survival Decision-Making: Within the threshold probability range of 10% to 50%, the net benefit of our model remained stable at 0.05 to 0.10. The model demonstrated its decision-making advantage in long-term survival assessment.

(3) 5-Year Survival Decision-Making: Even in the scenario of long-term follow-up (5 years), our model consistently demonstrated a net benefit ranging from 0.04 to 0.20 within a threshold probability of 15% to 50%. The results suggest that the model continues to hold practical value in long-term clinical management.

In conclusion, the DCA results further confirm that our model not only outperforms previous models in predictive accuracy, as demonstrated by the C-index and AUC, but also provides higher net benefit in practical clinical decision-making. Especially in the assessment of short-to-medium-term (1-3 years) survival, it can offer more reliable decision-making basis for risk-stratified interventions in TNBC patients-such as prioritizing immunotherapy for high-risk patients and avoiding unnecessary or excessive chemotherapy for low-risk patients.

### Associations of the macrophage-based prognostic model with more advanced disease status

**Figure 4:**
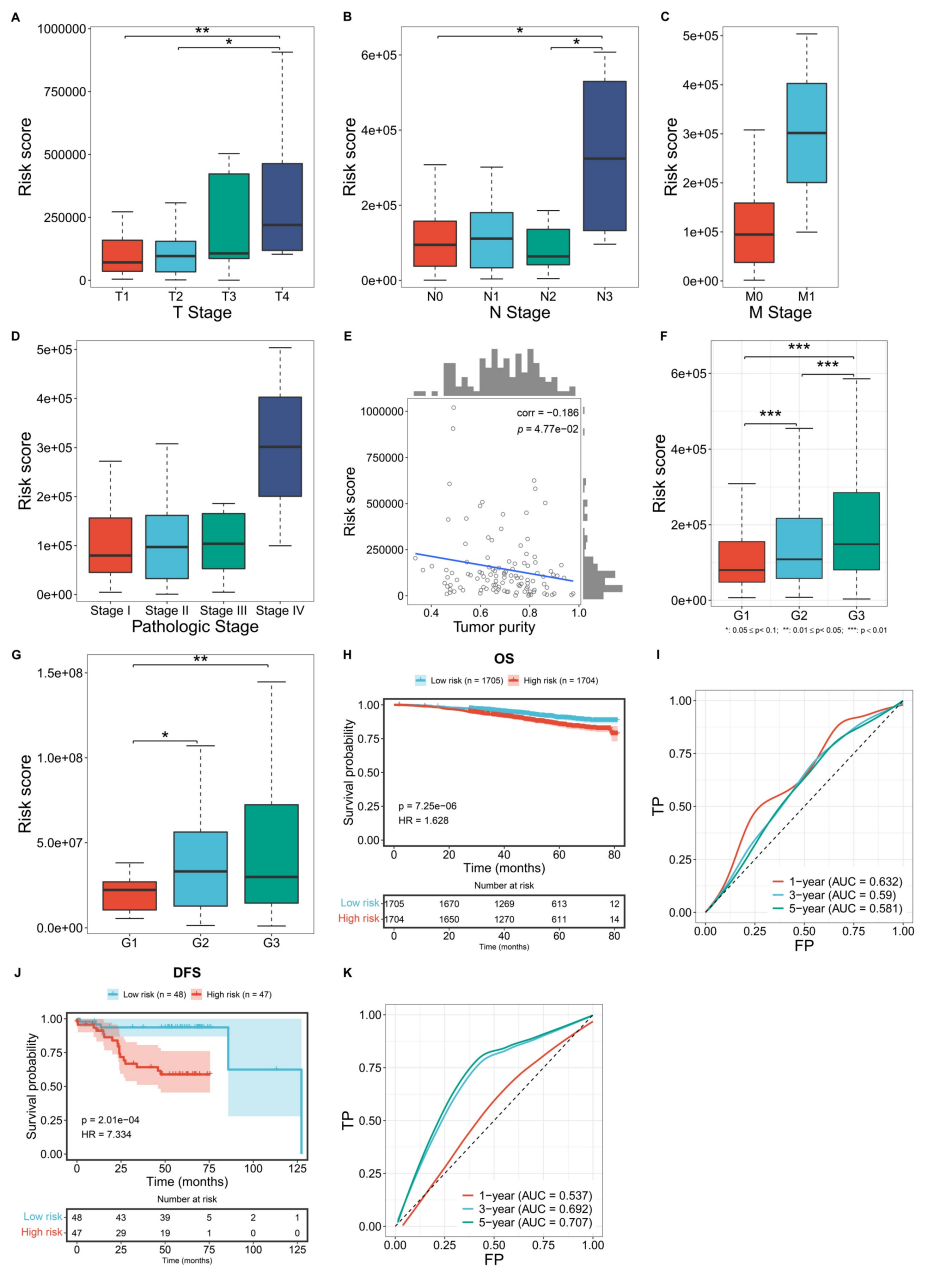
Associations of the macrophage-based prognostic model with clinicopathological traits and external validation of the model. (A-C) Distribution of the risk score across diverse T, N, M stages in the TCGA TNBC cohort. (D) Distribution of the risk score across different pathological stages of TCGA TNBC cases. (E) Correlation between the risk score and tumor purity among TCGA TNBC cases. (F, G) Distribution of the risk score across different histological grades in the GSE96058 and GSE45255 cohort. (H) OS probability of low- or high-risk patients in the GSE96058 cohort. (I) One-, three-, or five-year ROCs based on the model among the GSE96058 samples. (J) DFS probability of low- or high-risk patients in the GSE45255 cohort. (K) One-, three-, or five-year ROCs based on the model among the GSE45255 samples.

Further analysis was conducted to evaluate the correlations between the macrophage-based prognostic model and TNBC clinicopathological traits. TCGA patients with more advanced T, N, M stages, and pathological stages, exhibited significantly higher risk scores (**Figure 4A-D**). In addition, the risk score was negatively correlated with tumor purity (**Figure 4E**). In both the GSE96058 and GSE45255 cohorts, patients with more advanced histological grades exhibited prominently higher risk scores (**Figure 4F, G**). Overall, the macrophage-based prognostic model was associated with more advanced status of TNBC patients.

### External validation of the macrophage-based prognostic model

The GSE96058 and GSE45255 cohorts were used to independently validate the efficacy of the macrophage-relevant signature in predicting patient survival. In the GSE96058 cohort, high-risk cases were shown to have poorer OS versus low-risk cases (**Figure 4H**). Also, the model accurately predicted one-year survival (**Figure 4I**). In the GSE45255 cohort, shorter DFS time was investigated in high-risk cases, with the reliable efficacy in DFS prediction (**Figure 4J, K**). These results confirm the generalizability of the macrophage-based prognostic model.

### Heterogeneous somatic mutations between low- and high-risk TNBC patients

**Figure 5:**
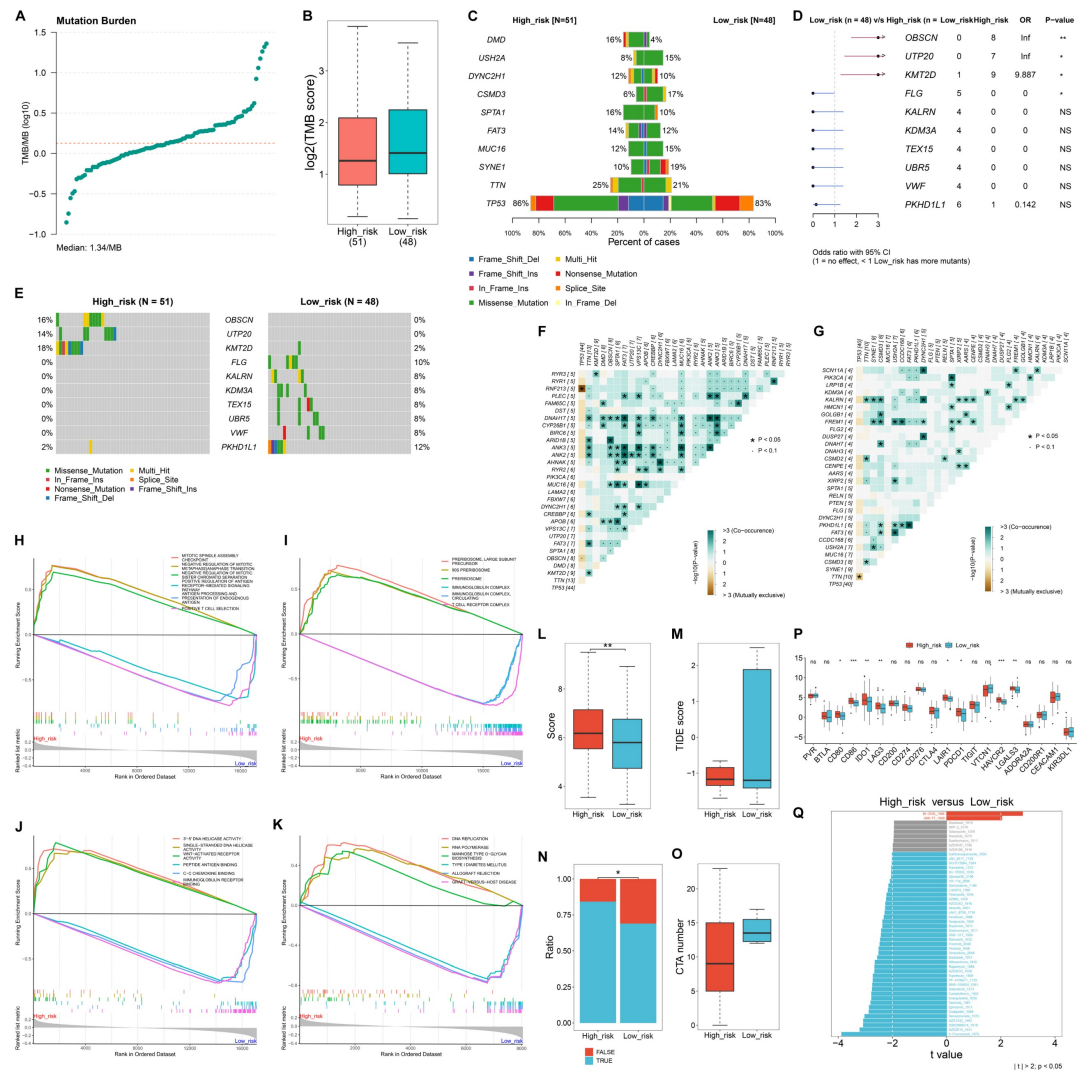
Heterogeneous somatic mutations, molecular mechanisms, and responses to immunotherapy between low- and high-risk TNBC patients. (A) Distribution of TMB scores across TNBC samples. (B) Comparison of TMB scores in high- versus low-risk groups. (C) The top ten mutated genes in low- and high-risk samples. (D, E) Comparison of the frequency of mutated genes in low- or high-risk group. (F) Co-occurring or mutually exclusive mutated genes in high-risk samples. (G) Co-occurring or mutually exclusive mutated genes in low-risk samples. (H-K) GSEA showing the main (H) biological processes, (I) cellular components, (J) molecular functions, and (K) KEGG pathways that were significantly different between high- and low-risk groups. (L, M) Comparison of (L) T-cell inflamed scores and (M) TIDE scores between low- and high-risk TCGA TNBC patients. (N) Distribution of the ratio of responders or non-responders to immunotherapy in low- or high-risk group. (O) Difference in CTA numbers between groups. (P) Comparison of the expression of immune checkpoints between groups. (Q) Differences in the sensitivity to drugs between groups. NS: no significance; *p≤0.05; **p≤0.01; ***p≤0.001.

The study quantified TMB score across TNBC samples, with the median TMB of 1.34/MB (**Figure 5A**). Overall, low-risk samples occurred relatively higher TMB scores versus high-risk samples (**Figure 5B**). TP53 had the highest mutation frequency in both low- and high-risk samples (**Figure 5C**). OBSCN, UTP20, and KMT2D had significantly higher mutation frequencies in high-risk cases compared to low-risk ones, whereas FLG had a significantly lower mutation frequency in low- versus high-risk cases (**Figure 5D, E**). In addition, co-occurring mutations were more frequently observed in high-risk samples than in low-risk samples (**Figure 5F, G**).

### Molecular mechanisms underlying the macrophage-based prognostic model

In terms of biological processes, mitotic spindle assembly checkpoint, negative regulation of mitotic metaphase anaphase transition, and mitotic sister chromatid separation were highly enriched in high-risk samples, while positive regulation of antigen receptor-mediated signaling pathway, and antigen processing and presentation of endogenous antigen, and positive T cell selection were prominently enriched in low-risk samples (**Figure 5H**). Regarding cellular components, preribosome, large subunit precursor, and preribosome were highly enriched in high-risk group, while the low-risk group showed significant enrichment in immunoglobulin complex and T cell receptor complex (**Figure 5I**). For molecular functions, 3'-5' DNA helicase activity, single-stranded DNA helicase activity, and WNT-activated receptor activity were enriched in high-risk cases. In contrast, peptide antigen binding, C-C chemokine binding, and immunoglobulin receptor binding were prominently enriched in low-risk cases (**Figure 5J**). Moreover, for KEGG pathways, DNA replication, RNA polymerase, and mannose type O-glycan biosynthesis were notably enriched in the high-risk group, whereas type I diabetes mellitus, allograft rejection, and graft-versus-host disease pathways were significantly enriched in the low-risk group (**Figure 5K**).

### High-risk patients exhibit a stronger response to immunotherapy

High-risk group exhibited a higher T-cell inflamed score and a lower TIDE score in comparison to low-risk group (**Figure 5L, M**). In addition, a greater proportion of responders to immunotherapy was observed in the high-risk group versus the low-risk group (**Figure 5N**). In **Figure 5O**, CTA number was remarkably higher the low-risk group than in the high-risk group. Most immune checkpoints, including CD80, CD86, IDO1, LAG3, LAIR1, PDCD1, HAVCR2, and LGALS3, showed the notably higher expression in the high-risk group compared to the low-risk group (**Figure 5P**). These findings suggest that high-risk patients may have a better response to immunotherapy.

### Heterogeneous drug sensitivity between low- and high-risk TNBC patients

Low-risk samples exhibited significantly lower IC50 values for BI-2536 BI-2536, and UMI-77, indicating greater sensitivity to these drugs (**Figure 5Q**). In contrast, high-risk individuals showed increased sensitivity to a range of other drugs, including 5-Fluorouracil, AZD2014, GSK2606414, AZD1332, Temozolomide, Oxaliplatin, Epirubicin, Taselisib, Entospletinib, Camptothecin, Dabrafenib, BMS-536924, PF-4708671, Topotecan, AZD8055, Rapamycin, Mitoxantrone, Dactolisib, Vinorelbine, Pictilisib, Foretinib, Ribociclib, GNE-317, Dactinomycin, Buparlisib, Teniposide, Irinotecan, JAK1_8709, Alisertib, AZD5363, AZ960, Palbociclib, LGK974, Gemcitabine, VX-11e, Uprosertib, KU-55933, Trametinib, SCH772984, JAK_8517, and Gallibiscoquinazole.

### Experimental verification of the expression of signature genes

**Figure 6:**
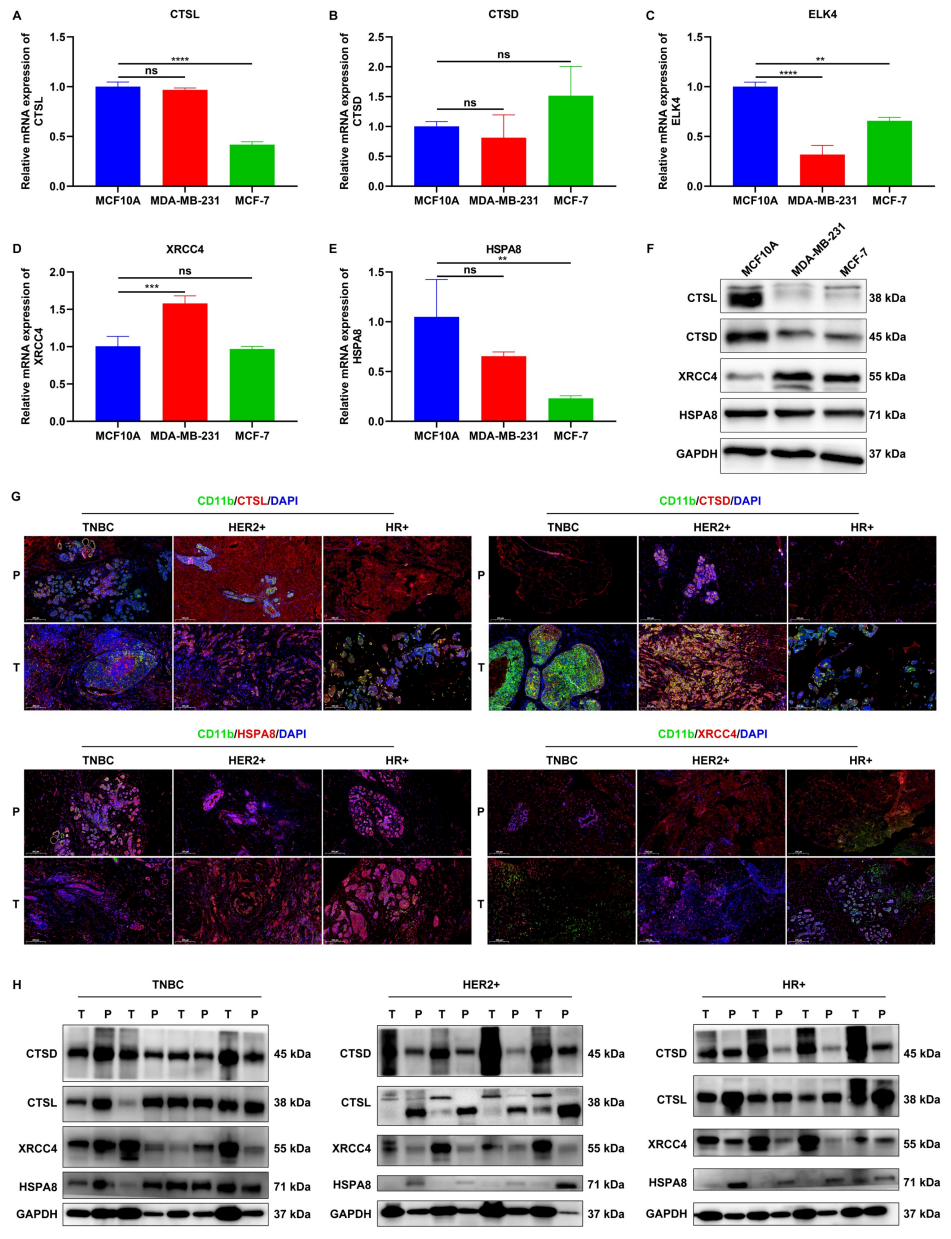
Experimental verification of the expression of signature genes. (A-E) RT-qPCR of the mRNA expression of CTSL, CTSD, ELK4, XRCC4, and HSPA8 in MCF10A, MDA-MB-231, and MCF-7 cells. (F) Western blot analysis of CTSL, CTSD, XRCC4, and HSPA8 protein expression in MCF10A, MDA-MB-231, and MCF-7 cells. (G) Representative IF images showing CD11b+CTSL+, CD11b+CTSD+, CD11b+XRCC4+, and CD11b+HSPA8+ cells in tumor (T) tissues and paracancerous (P) normal tissues from patients with TNBC (n=5), HER2^+^ breast cancer (n=5), and HR^+^ breast cancer (n=5). Scale bar: 200 μm. CD11b, green; CTSL/CTSD/ELK4/XRCC4/HSPA8, red. (H) Western blot analysis of CTSL, CTSD, XRCC4, and HSPA8 expression in tumor tissues and paracancerous normal tissues from patients with TNBC (n=5), HER2^+^ breast cancer (n=5), and HR^+^ breast cancer (n=5). ns: no significance; **p≤0.01; ***p≤0.001; ****p≤0.0001.

We validated the expression of signature genes in mammary epithelial cells (MCF10A) and two breast cancer cell lines (MDA-MB-231, and MCF-7). RT-qPCR results showed that CTSL expression was significantly lower in MCF-7 cells, but not in MDA-MB-231 cells compared to MCF10A cells (**Figure 6A**). No significant difference in CTSD expression was detected between MCF10A cells and either MDA-MB-231 or MCF-7 cells (**Figure 6B**). In comparison to MCF10A cells, lower ELK4 expression was found in both MDA-MB-231 and MCF-7 cells (**Figure 6C**). XRCC4 expression was remarkably higher in MDA-MB-231 cells, but in not MCF-7 cells, relative to MCF10A cells (**Figure 6D**). In addition, HSPA8 was found to be remarkably lower in MCF-7 cells, but not in MDA-MB-231 cells compared to MCF10A cells (**Figure 6E**). Western blot results demonstrated that the expression of CTSL and CTSD was notably down-regulated, while XRCC4 expression was notably up-regulated both in MDA-MB-231 and MCF-7 cells relative to MCF10A cells (**Figure 6F**). HSPA8 expression was modestly down-regulated in both cancer cell lines compared to MCF10A cells. However, ELK4 expression was undetectable in all three cell lines.

To further explore the clinical relevance, we collected tumor (T) tissues and paracancerous (P) normal tissues from patients with TNBC (n=5), HER2^+^ breast cancer (n=5), and HR^+^ breast cancer (n=5). Immunofluorescence assays revealed abnormal expression of CTSL, CTSD, HSPA8, and XRCC4 in CD11b^+^ cells in tumor tissues than those in paracancerous normal tissues, regardless of breast cancer subtypes (**Figure 6G**). Furthermore, western blot also confirmed the aberrant expression of CTSL, CTSD, HSPA8, and XRCC4 in tumor tissues relative to paracancerous normal tissues (**Figure 6H**). These findings suggest that these signature genes may play a role in the development and progression of breast cancer.

### Knockdown of XRCC4 impairs the malignant behaviors of MCF10A, MDA-MB-231, and MCF-7 cells

**Figure 7:**
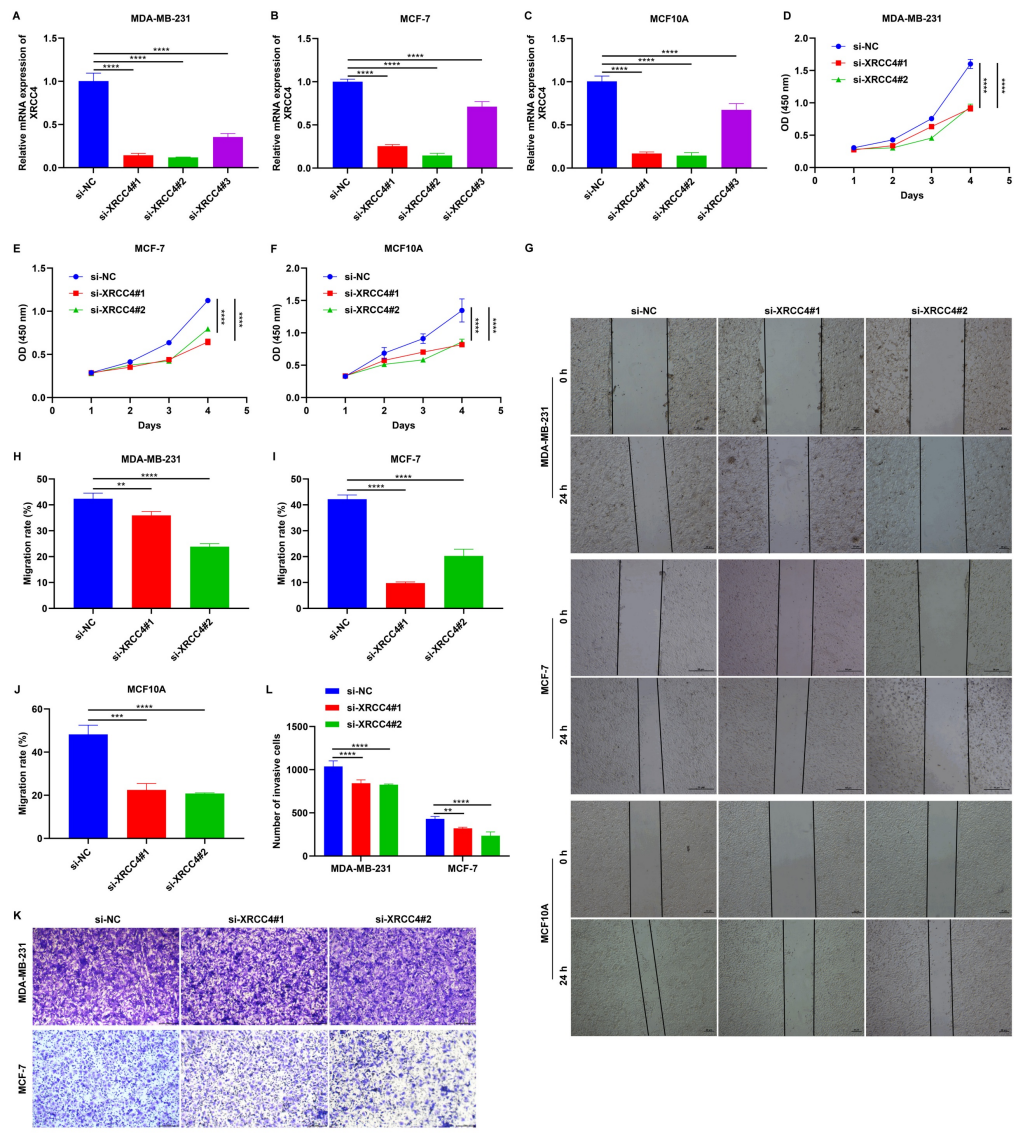
Knockdown of XRCC4 attenuates malignant behaviors of breast cancer cells. (A-C) RT-qPCR of the mRNA expression of XRCC4 in MCF10A, MDA-MB-231, and MCF-7 cells transfected with XRCC4 siRNAs or controls. (D-F) CCK-8 results for (D) MCF10A, (E) MDA-MB-231, and (F) MCF-7 cells with XRCC4 siRNAs or controls. (G-J) Wound scratch assay results for MCF10A, MDA-MB-231, and MCF-7 cells with XRCC4 siRNAs or controls. Scale bar: 20, or 50 μm. (K, L) Transwell assay of MDA-MB-231, and MCF-7 breast cancer cells with XRCC4 siRNAs or controls. Scale bar: 200 μm. **p≤0.01; ***p≤0.001; ****p≤0.0001.

To investigate the role of XRCC4 in tumor cell migration, MCF10A, MDA-MB-231, and MCF-7 cells were transfected with specific siRNAs of XRCC4. As a result, XRCC4 expression was significantly lowered in all three cell lines, particularly with si-XRCC4#1 and si-XRCC4#2 (**Figure 7A-C**). CCK-8 and wound scratch assays showed that cells transfected with si-XRCC4#1 and si-XRCC4#2 exhibited significantly lower proliferative (**Figure 7D-F**), and migratory (**Figure 7G-J**) capacities in comparison to those transfected with si-NC. In addition, Transwell results showed that silencing XRCC4 impaired the invasive capacities of MDA-MB-231 and MCF-7 breast cancer cells (**Figure 7K, L**). Therefore, silencing XRCC4 attenuates malignant behaviors of TNBC cells.

### Overexpression of CTSL, CTSD, and HSPA8 impair malignant behaviors of MCF10A, MDA-MB-231, and MCF-7 cells

**Figure 8:**
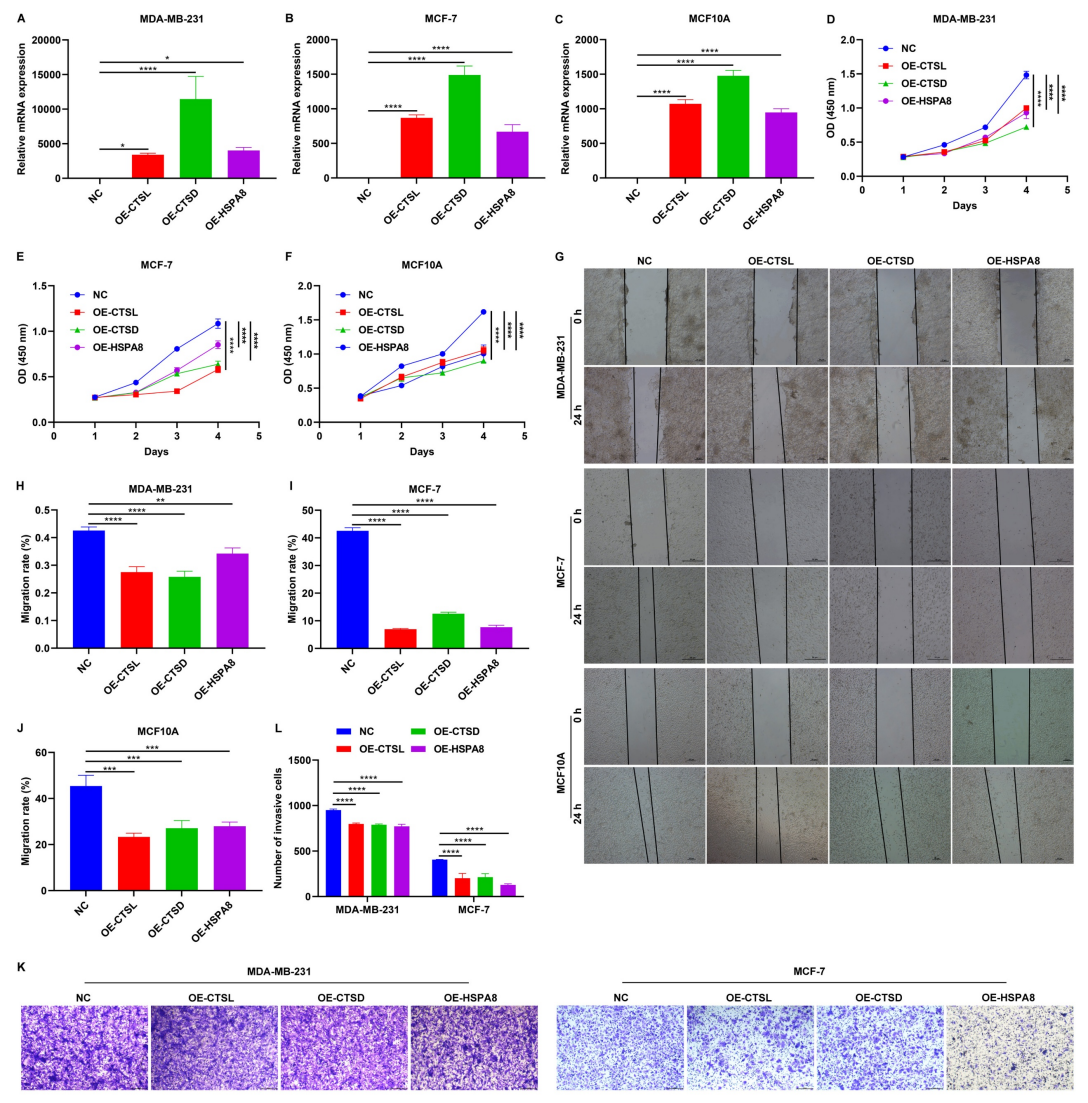
Overexpression of CTSL, CTSD, and HSPA8 mitigates malignant behaviors of breast cancer cells. (A-C) RT-qPCR of the mRNA expression of CTSL, CTSD, and HSPA8 in MCF10A, MDA-MB-231, and MCF-7 cells transfected with the corresponding gene overexpression plasmids. (D-F) CCK-8 results for (D) MCF10A, (E) MDA-MB-231, and (F) MCF-7 cells transfected with empty vectors or CTSL, CTSD or HSPA8 overexpression plasmids. (G-J) Wound scratch assay results for MCF10A, MDA-MB-231, and MCF-7 cells transfected with empty vectors or CTSL, CTSD or HSPA8 overexpression plasmids. Scale bar: 20, or 50 μm. (K, L) Transwell assay of MDA-MB-231, and MCF-7 breast cancer cells transfected with empty vectors or CTSL, CTSD or HSPA8 overexpression plasmids. Scale bar: 200 μm. *p≤0.05; **p≤0.01; ***p≤0.001; ****p≤0.0001.

The expression of CTSL, CTSD, and HSPA8 were significantly increased following the transfection of corresponding gene overexpression plasmids in MCF10A, MDA-MB-231, and MCF-7 cells (**Figure 8A-C**). As demonstrated by CCK-8 and wound scratch assay results, overexpression of CTSL, CTSD, and HSPA8 significantly attenuated the proliferative (**Figure 8D-F**) and migratory (**Figure 8G-J**) capacities of these TNBC cells. In addition, Transwell assay showed that the invasive capacity of TNBC cells was also impaired by the overexpression of CTSL, CTSD, and HSPA8 (**Figure 8K, L**). Thus, CTSL, CTSD, and HSPA8 can reduce the malignant behaviors of TNBC cells.

## Discussion

TNBC is an aggressive subtype characterized by extensive intratumoral heterogeneity^38^. Recent technological advances have enabled more reliable and integrative single-cell analysis of the tumor microenvironment at the transcriptional level^39^,^40^, facilitating the observation of cell populations and cell-cell crosstalk^41^. In this work, we combined scRNA-seq with bulk transcriptome data to systematically analyze the cellular components within the TNBC tumor microenvironment. Tumorigenesis is governed both by genetically altered tumor cells and non-malignant host cells within the tumor microenvironment, which significantly affect tumor progression, metastasis, and therapeutic outcomes^42^. We reported that the TNBC microenvironment comprises B cells, dendritic cells, endothelial cells, epithelial cells, fibroblasts, macrophages, monocytes, plasmablasts, and T cells. Most of them (B cells, dendritic cells, fibroblasts, macrophages, plasmablasts, and T cells) presented at higher abundance in TNBC versus non-TNBC tissues. Bulk transcriptome analysis further confirmed a significant enrichment of macrophages in the TNBC microenvironment. In addition, we generated two maps of cellular interactions within the microenvironments of non-TNBC and TNBC. Our results revealed more active cell-cell crosstalk in the TNBC microenvironment, especially macrophages with other cell populations, highlighting the crucial implication of macrophages in TNBC.

Inter-patient and intra-tumor heterogeneity pose significant challenges in identifying predictive biomarkers and developing effective treatments for TNBC^43^. This work proposed a novel macrophage-based prognostic model comprising five genes: CTSD, CTSL, ELK4, HSPA8, and XRCC4. A high risk score was predictive of poor prognostic outcomes. External validation confirmed that the model reliably predicts TNBC patient prognosis, especially one-year survival. Somatic mutations were extensively heterogeneous between low- and high-risk TNBC patients. Additionally, high-risk patients demonstrated greater responsiveness to immunotherapy, as indicated by higher T-cell inflamed scores, lower TIDE scores, and up-regulated immune checkpoint molecules, such as CD80, CD86, IDO1, LAG3, LAIR1, PDCD1, HAVCR2, and LGALS3. We also investigated the heterogeneous sensitivity to drugs between low- and high-risk patients. Thus, the macrophage-relevant gene signature displayed the potential in estimating both prognostic outcomes and therapy response in TNBC. Mechanistically, signature genes link macrophage function to TNBC progression: XRCC4 (DNA repair) promotes immune evasion via STING pathway suppression; CTSL/CTSD (cathepsins) regulate microenvironment remodeling; HSPA8 (chaperone) modulates stress responses. Their aberrant expression and functional roles were validated *in vitro*, underscoring their potential as therapeutic targets.

High XRCC4 expression is associated with poor progression-free survival following radiotherapy for TNBC patients.* In vitro* studies have shown that silencing XRCC4 sensitizes TNBC cells to ionizing radiation^44^. Herein, XRCC4 was proven to be up-regulated in TNBC cells versus healthy cells. Knockdown of XRCC4 impaired malignant behaviors of TNBC cells. Nuclear CTSL has been identified as a positive biomarker of TNBC^45^. BRCA1 deficiency activates CTSL-induced degradation of 53BP1 in TNBC cells. The nuclear levels of CTSL, vitamin D receptor, and 53BP1 act as a triple biomarker signature for stratifying patients with BRCA1-mutated tumors and TNBC, providing predictive value for drug response. Elevated CTSD expression correlates with poor recurrence-free survival of TNBC patients, and extracellular CTSD can be measured within the tumor microenvironment, but not in normal breast stroma^46^. A 9-kDa matricellular SPARC fragment released by CTSD displays pro-tumor activity in the TNBC microenvironment^47^. Immunotherapy with CTSD-targeting antibody has been emerged as a promising therapeutic approach against TNBC. HSPA8 has also been linked to TNBC survival.^48^. CTSL, CTSD, and HSPA8 were down-regulated in breast cancer cells, and their overexpression attenuated malignant behaviors of TNBC cells. Altogether, the aberrantly expressed signature genes (XRCC4, CTSL, CTSD, and HSPA8) is closely related to tumor malignant progression.

Nevertheless, closely related experiments are needed to verify the roles of the signature genes in TNBC malignant behaviors. In addition, the efficacy of the macrophage-based gene signature in survival prediction requires to be validated in larger prospective cohorts.

## Conclusion

Collectively, based on the integration of single-cell and bulk transcriptome analyses, this study proposes a novel macrophage-based gene signature - comprising CTSD, CTSL, ELK4, HSPA8, and XRCC4 - for prediction of survival outcomes and therapy responses in TNBC patients. The signature genes were shown to be associated with tumor malignant behaviors. These findings may positively influence future clinical research involving macrophages in TNBC.

## Supplementary Material

Supplementary figure 1. Quality control of scRNA-seq data. (A-L) Removal of empty droplets and single cells with a mitochondrial gene proportion greater than 10% and a ribosomal gene proportion less than 10% in (A-C) GSM5457199; (D-F) GSM5457205; (G-I) GSM5457208; and (J-L) GSM5457211 specimens. Supplementary figure 2. PCA of scRNA-seq data after quality control. (A) The top two PCs. (B) Selection of the optimal number of PCs. (C) PCA plots of single cells from TNBC and non-TNBC. (D) The top 20 marker genes associated with the top nine PCs. Supplementary figure 3. Single-cell clustering analysis. (A) UMAP for clustering single cells into distinct groups. (B) Distribution of TNBC and non-TNBC single cells. (C) Comparison of cell ratios across identified clusters between TNBC and non-TNBC. (D) Top 10 marker genes identified for each cell cluster. (E) The top one marker gene for each cell cluster.

Supplementary table 1. Differentially expressed transcription factors in TNBC macrophages versus non-TNBC macrophages.

Supplementary table 2. Up-regulated genes in TNBC macrophages versus non-TNBC macrophages.

Supplementary table 3. Down-regulated genes in TNBC macrophages versus non-TNBC macrophages.

## Abbreviations

TNBC: triple-negative breast cancer
HER2: human epidermal growth factor receptor
scRNA-seq: single-cell RNA sequencing
GEO: Gene Expression Omnibus
TCGA: The Cancer Genome Atlas
PCA: principal component analysis
PCs: principal components
UMAP: uniform manifold approximation and projection
FC: fold change
GO: Gene Ontology
KEGG: Kyoto Encyclopedia of Genes and Genomes
GSEA: gene set enrichment analysis
DEGs: differentially expressed genes
LASSO: least absolute shrinkage and selection operator
CTA: cancer-testis antigen
siRNAs: small interfering RNAs
HR: hormone receptor
IF: immunofluorescence
CCK-8: Cell Counting Kit-8
OS: overall survival
DFS: disease-free survival
ROCs: receiver operator characteristic curves
