# Supplementary figures and images for: Integration of Single-cell and Bulk Transcriptome Analyses Unravels a Macrophage-based Gene Signature for Prognostication and Treatment in Triple-negative Breast Cancer
Source: Int J Med Sci. 2026 Jan 1;23(1):253–70. doi: 10.7150/ijms.120593 (PMC12702127; doi:10.7150/ijms.120593)

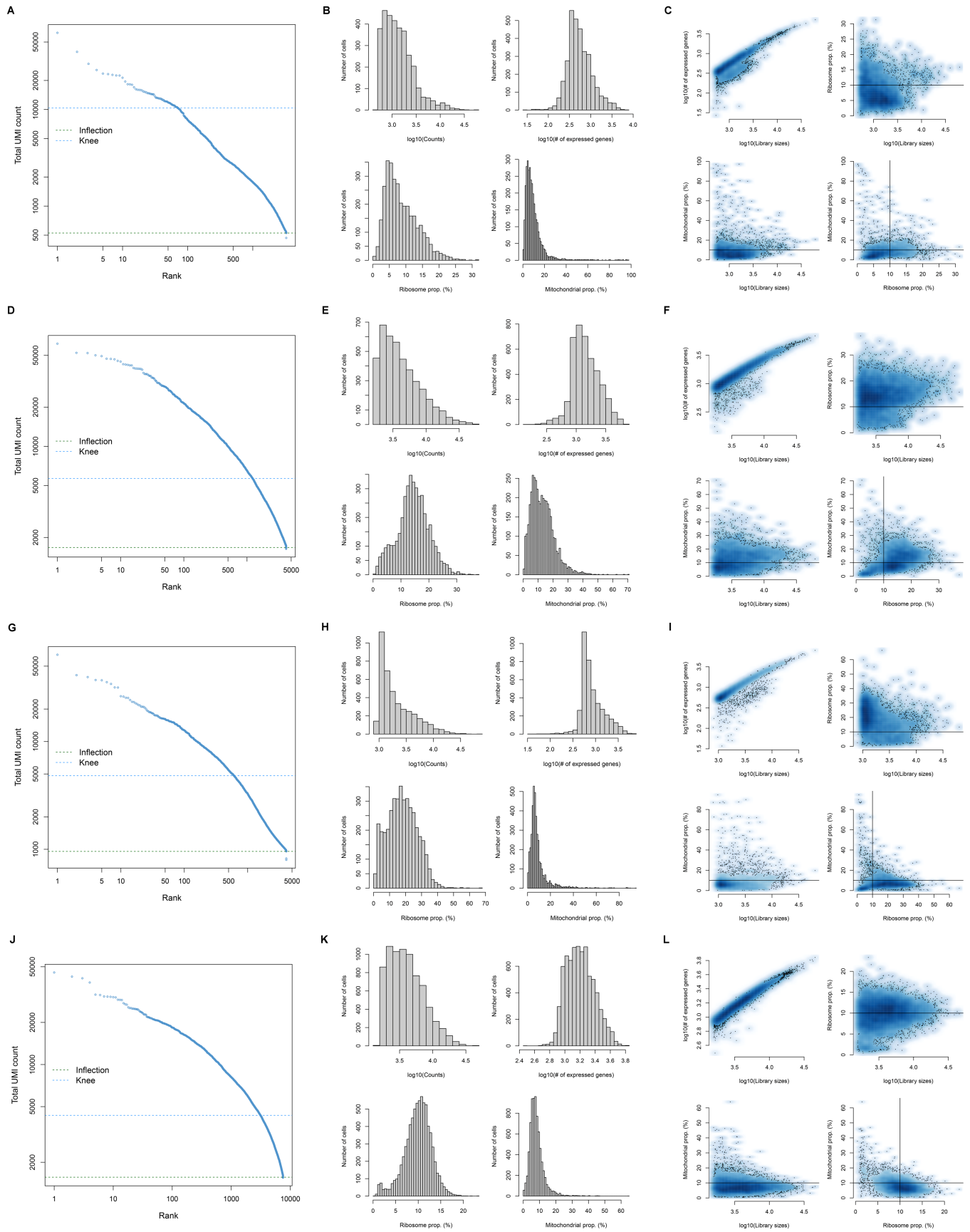

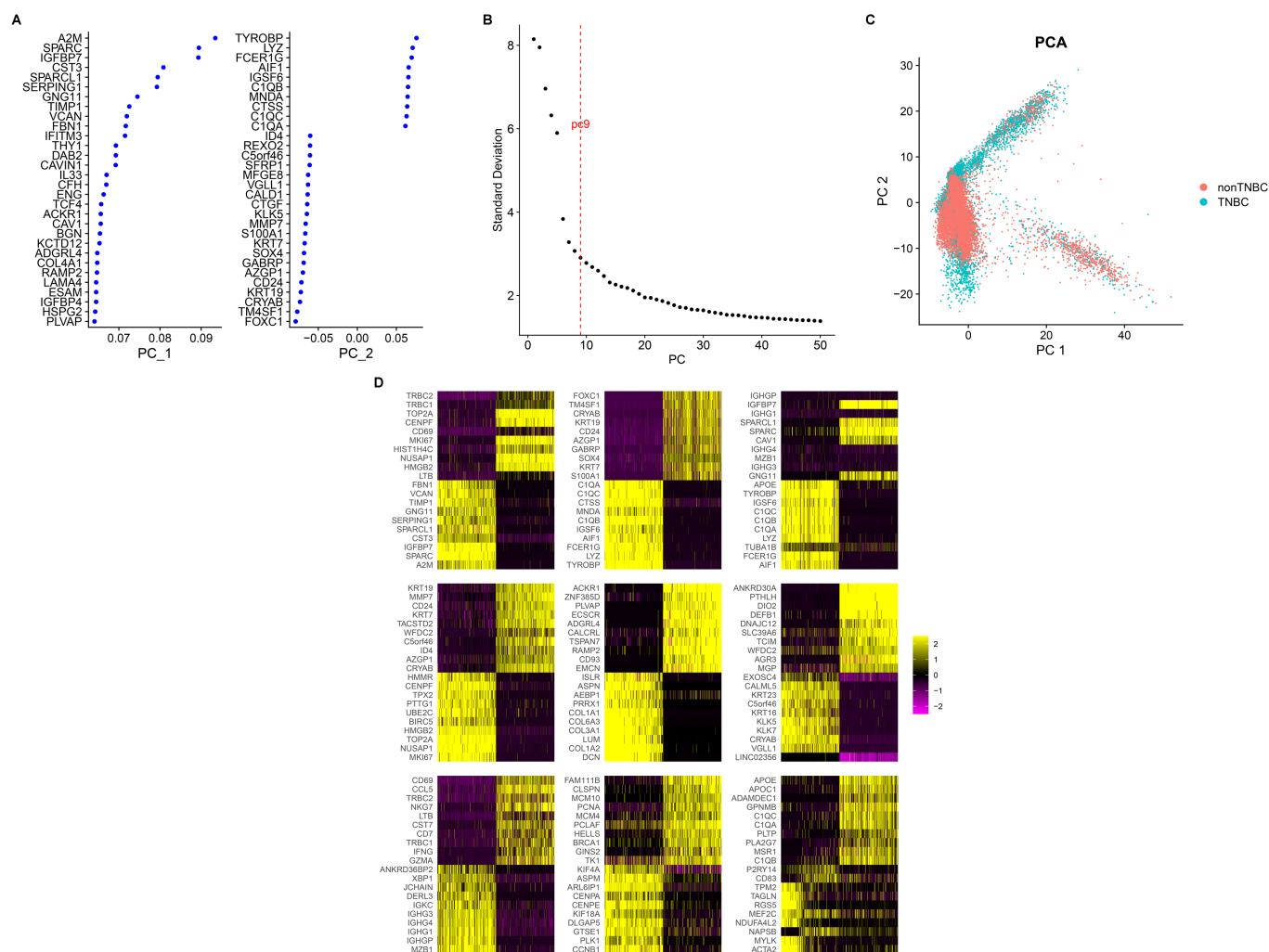

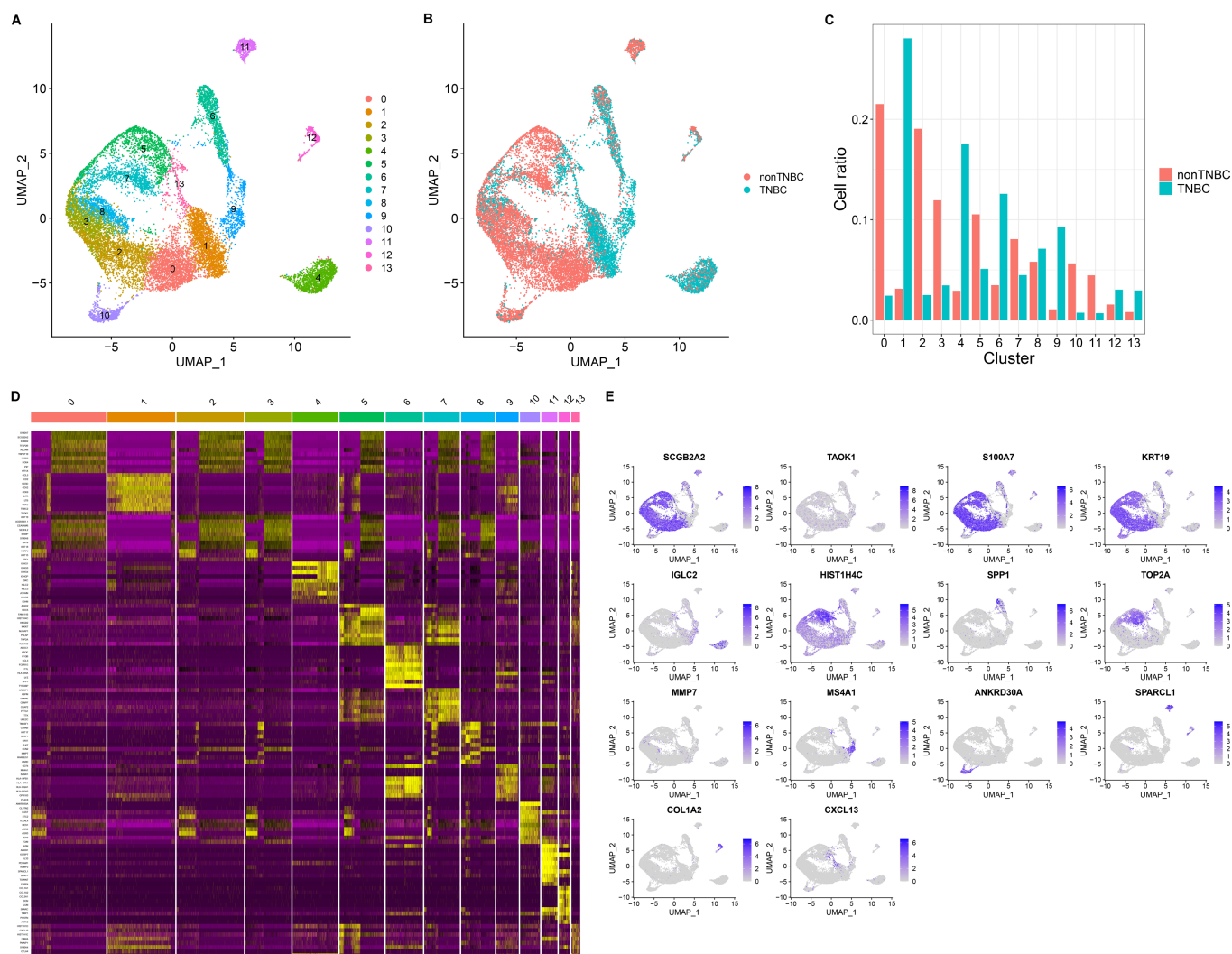

Supplement: Supplementary file 1 — Supplementary figure 1. Quality control of scRNA-seq data. (A-L) Removal of empty droplets and single cells with a mitochondrial gene proportion greater than 10% and a ribosomal gene proportion less than 10% in (A-C) GSM5457199; (D-F) GSM5457205; (G-I) GSM5457208; and (J-L) GSM5457211 specimens. Supplementary figure 2. PCA of scRNA-seq data after quality control. (A) The top two PCs. (B) Selection of the optimal number of PCs. (C) PCA plots of single cells from TNBC and non-TNBC. (D) The top 20 marker genes associated with the top nine PCs. Supplementary figure 3. Single-cell clustering analysis. (A) UMAP for clustering single cells into distinct groups. (B) Distribution of TNBC and non-TNBC single cells. (C) Comparison of cell ratios across identified clusters between TNBC and non-TNBC. (D) Top 10 marker genes identified for each cell cluster. (E) The top one marker gene for each cell cluster. [file ijmsv23p0253s1.pdf]
